# The complete chloroplast genome sequence of *Cosmos bipinnatus*, the first of the genu*s Cosmos*

**DOI:** 10.1080/23802359.2019.1693298

**Published:** 2019-11-22

**Authors:** Mei Jiang, Haimei Chen, Liqiang Wang, Jinwen You, Chang Liu

**Affiliations:** a Key Laboratory of Bioactive Substances and Resource Utilization of Chinese Herbal Medicine from Ministry of Education, Engineering Research Center of Chinese Medicine Resources from Ministry of Education, Institute of Medicinal Plant Development, Chinese Academy of Medical Sciences and Peking Union Medical College, Beijing, P. R. China;; b Institute of Chinese Herbal Medicine, Hubei Academy of Agricultural Sciences, Enshi, Hubei, P. R. China

**Keywords:** *Cosmos bipinnatus*, chloroplast genome, phylogeny

## Abstract

*Cosmos bipinnatus* has been used widely in traditional medicines. Here, we sequenced and assembled the complete chloroplast genome of *C. bipinnatus*. This genome is 150,356 bp in size with a pair of inverted repeats (IRs) of 25,082 bp, a large single-copy (LSC) region of 83,003 bp, and a small single-copy region (SSC) of 18,397 bp. It contains 112 unique genes, including 80 protein-coding, 4 rRNA, and 28 tRNA genes. The phylogenomic analysis showed the *C. bipinnatus* and species belonging to the Madieae, Millerieae, Heliantheae, and Eupatorieae tribes were clustered together. The availability of chloroplast genome provided valuable information for future conservation, taxonomy, evolution, and differentiation studies of *C. bipinnatus.*

*Cosmos bipinnatus*, commonly called the garden cosmos or Mexican aster, belongs to the *Cosmos* genus, Asteraceae family (NCBI:txid51277 2019). Chemical components with diverse structures, including monoterpenes (Olajuyigbe and Ashafa 2014), sesquiterpene (Sohn et al. 2013), triterpene alcohols (Akihisa et al. 1996), and flavonoids (Saito 1974) had been isolated from *C. bipinnatus*. Pharmacological studies indicated the extracts of *C. bipinnatus* has anti-inflammatory activity by inhibiting the expression of inducible nitric oxide synthase, cyclooxygenase-2, and pro-inflammatory cytokines (Sohn et al. 2013). In contrast, there is little research on the molecular genetics of *C. bipinnatus*, which limited the resource conservation, species identification, and drug development of *C. bipinnatus*. In this study, we reported the first complete chloroplast genome sequence of *C. bipinnatus*.

Fresh leaf samples were collected from the Central China Medicinal Botanical Garden, Enshi, Hubei China (E30°17′84″, N109°74′39″) and identified as from *C. bipinnatus* by Professor Jinwen You. The genomic DNA was extracted with plant genomic DNA kit (Tiangen Biotech, Beijing, China), the genome sequence was completed using the Hiseq 2500 platform (Illumina, San Diego, CA, USA). A voucher specimen and its DNA (accession number: 201808281) were deposited at Institute of Medicinal Plant Development. The raw sequence data were assembled into a chloroplast genome by NOVOPlasty (version 2.7.2) (Dierckxsens et al. 2017). CpGAVAS2 was used to annotate the chloroplast genome (Shi et al. 2019).

The chloroplast genome of *C. bipinnatus* (GenBank accession number: MN518845) is 150,356 bp in size with a pair of inverted repeats (IRs) of 25,082 bp separated by a large single-copy (LSC) region of 83,003 bp and a small single-copy (SSC) region of 18,397 bp. The chloroplast genome encoded 129 genes, of which 112 are unique genes including 80 protein-coding, 4 ribosome RNA (rRNA), and 28 transfer RNA (tRNA) genes. Among them, seven protein-coding genes had one intron and two protein-coding genes had two introns. Six tRNA genes were found to contain one intron. The GC content of the whole genome was 37.57%, of which the protein-coding, the rRNA, and the tRNA genes were 37.83, 54.67, and 53.15%, respectively. Within the protein-coding regions, the GC contents for the first, second and third positions of the codons were 45.53, 38.04, and 29.92%, respectively.

We obtained 51 complete chloroplast genome sequences to explore the phylogenomic relationships among the species from the Asteroideae subfamily. A total of 57 shared proteins present among these chloroplast genomes were subjected to multiple sequence alignment using CLUSTALW2 (version 2.0.12) (Thompson et al. 2002). Then, the phylogenomic tree was constructed using the maximum likelihood method implemented in RaxML (version 8.2.4) (Stamatakis 2014). As shown (Figure 1), the 11 tribes were divided into four branches. All species from the same tribe were grouped together, consistent with the current taxonomic classification. The phylogenomic tree showed the *C. bipinnatus* and species belonging to the Madieae, Millerieae, Heliantheae, and Eupatorieae tribes were clustered together with a support value of 83. The availability of chloroplast genome provided valuable information for future conservation, taxonomy, evolution, and differentiation studies of *C. bipinnatus*.

**Figure 1.**
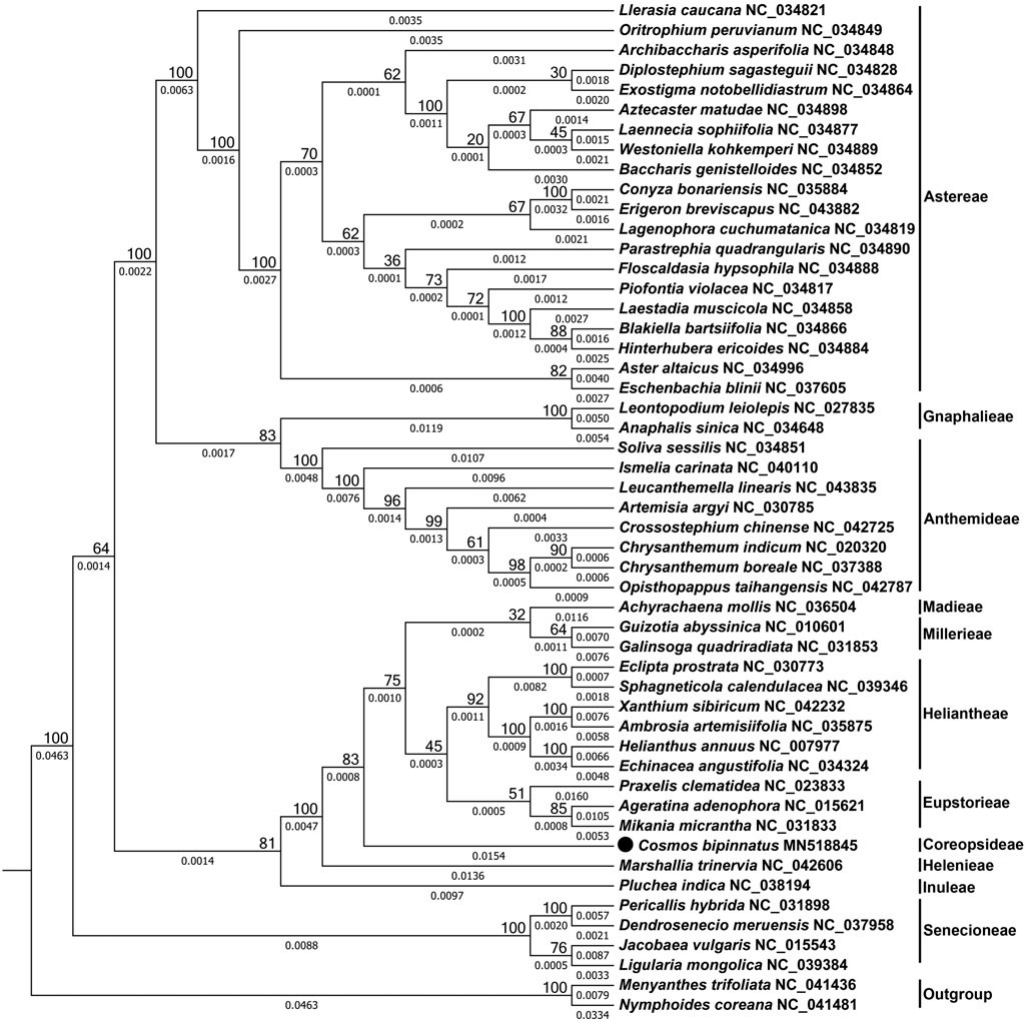
The phylogenomic tree of species from Asteroideae subfamily constructed using the maximum likelihood (ML) method using the 57 shared proteins, RPS12, PSBA, MATK, PSBI, PSBM, RPOB, RPOC2, RPS2, ATPH, ATPF, ATPA, PSBD, PSAB, PSAA, NDHJ, NDHK, NDHC, ATPE, ATPB, RBCL, PSAI, YCF4, CEMA, PETA, PSBF, PSBE, PETL, PETG, RPL33, RPS18, RPL20, PSBT, PSBN, PSBH, PETB, PETD, RPS11, RPL36, INFA, RPS8, RPL14, RPL16, RPS3, RPL23, NDHB, RPS7, RPS15, NDHH, NDHA, NDHI, NDHG, NDHE, PSAC, NDHD, CCSA, RPL32, and NDHF. The number below each line represents the branch length, the number above each node represents are the bootstrap values. The GenBank accession number for the corresponding chloroplast sequences were shown after the Latin name of the species.
